# Exercise training-induced speeding of ${\dot{\text{V}}\text{O}}_{{2}}$ kinetics is not intensity domain-specific or correlated with indices of exercise performance

**DOI:** 10.1007/s00421-024-05674-1

**Published:** 2024-12-05

**Authors:** Erin Calaine Inglis, Letizia Rasica, Danilo Iannetta, Kate M. Sales, Daniel A. Keir, Martin J. MacInnis, Juan M. Murias

**Affiliations:** 1 Faculty of Kinesiology, University of Calgary, Calgary, AB Canada; 2 School of Kinesiology, Western University, London, ON Canada; 3 College of Health and Life Sciences, Hamad Bin Khalifa University, Doha, Qatar

**Keywords:** Cycling exercise, Oxygen uptake kinetics, Endurance training, Exercise intensity domains

## Abstract

**Purpose:**

This study examined the effect of 3 and 6 weeks of intensity domain-based exercise training on ${\dot{\text{V}}\text{O}}_{{2}}$ kinetics changes and their relationship with indices of performance.

**Methods:**

Eighty-four young healthy participants (42 M, 42 F) were randomly assigned to six groups (14 participants each, age and sex-matched) consisting of: continuous cycling in the (1) moderate (MOD)-, (2) lower heavy (HVY1)-, and (3) upper heavy-intensity (HVY2)- domain; interval cycling in the (4) severe-intensity domain (i.e., high-intensity interval training (HIIT), or (5) extreme-intensity domain (i.e., sprint-interval training (SIT)); or (6) control (CON). Training participants completed two three-week phases of three supervised sessions per week, with physiological evaluations performed at PRE, MID and POST intervention. All training protocols, except SIT, were work-matched.

**Results:**

There was a significant time effect for the time constant ($\tau {\dot{\text{V}}\text{O}}_{{2}}$) between PRE (31.6 ± 10.4 s) and MID (22.6 ± 6.9 s) (p < 0.05) and PRE and POST (21.8 ± 6.3 s) (p < 0.05), but no difference between MID and POST (p > 0.05) and no group or interaction effects (p > 0.05). There were no PRE to POST differences for CON (p < 0.05) in any variables. Despite significant increases in maximal ${\dot{\text{V}}\text{O}}_{{2}}$ (${\dot{\text{V}}\text{O}}_{{{\text{2max}}}}$), estimated lactate threshold (θ_LT_), maximal metabolic steady state (MMSS), and peak power output (PPO) for the intervention groups (p < 0.05), there were no significant correlations from PRE to MID or MID to POST between $\Delta \tau {\dot{\text{V}}\text{O}}_{{2}}$ and $\Delta {\dot{\text{V}}\text{O}}_{{{\text{2max}}}}$ (r = – 0.221, r = 0.119), ΔPPO (r = – 0.112, r = – 0.017), Δθ_LT_ (r = 0.083, r = 0.142) and ΔMMSS (r = – 0.213, r = 0.049)(p > 0.05).

**Conclusion:**

This study demonstrated that (i) the rapid speeding of ${\dot{\text{V}}\text{O}}_{{2}}$ kinetics was not intensity-dependent; and (ii) changes in indices of performance were not significantly correlated with $\Delta \tau {\dot{\text{V}}\text{O}}_{{2}}$.

## Introduction

The rate of adjustment in the primary component (phase II) of pulmonary oxygen uptake (${\dot{\text{V}}\text{O}}_{{2}}$) to a greater metabolic rate (i.e., ${\dot{\text{V}}\text{O}}_{{2}}$ kinetics) within the moderate intensity domain displays a mono-exponential profile that reflects the dynamics of muscle ${\dot{\text{V}}\text{O}}_{{2}}$ (Rossiter 2011). The time constant of this adjustment ($\tau {\dot{\text{V}}\text{O}}_{{2}}$) to an increase in workload is a key parameter that represents an integrated response consisting of both central (i.e., oxygen delivery) and peripheral (i.e., mitochondrial respiration) components of aerobic function. Slower ${\dot{\text{V}}\text{O}}_{{2}}$ kinetics (i.e., greater τ values) are commonly observed in disease and low-fitness states (Palange et al. 1995; Schalcher et al. 2003; George et al. 2018) and are associated with a greater reliance on substrate-level phosphorylation (Poole and Jones 2012), greater metabolic disturbances (Grassi et al. 2000) and, potentially, reduced tolerance to exercise compared to faster ${\dot{\text{V}}\text{O}}_{{2}}$ kinetics (Jones and Burnley 2009; Goulding et al. 2021). Typically, endurance trained individuals display faster ${\dot{\text{V}}\text{O}}_{{2}}$ kinetics (i.e., smaller τ values) compared to less active or sedentary individuals (Grey et al. 2015; George et al. 2018; Inglis et al. 2021b), and a negative correlation has been reported between maximal ${\dot{\text{V}}\text{O}}_{{2}}$ (${\dot{\text{V}}\text{O}}_{{{\text{2max}}}}$) and the speed of adjustment of ${\dot{\text{V}}\text{O}}_{{2}}$ (Powers et al. 1985; Zhang et al. 1991; Inglis et al. 2021b) to an upper limit, beyond which further speeding of ${\dot{\text{V}}\text{O}}_{{2}}$ kinetics becomes unlikely (Murias et al. 2014; Korzeniewski et al. 2018; Inglis et al. 2021b). Additionally, along with markers of cardiorespiratory fitness such as V̇O_2max_ and the critical intensity of exercise, fast ${\dot{\text{V}}\text{O}}_{{2}}$ kinetics has been suggested to be an important component of performance (Jones and Burnley 2009; Rossiter 2011; Goulding et al. 2021).

From an endurance exercise training perspective, the role of exercise intensity on the speeding of ${\dot{\text{V}}\text{O}}_{{2}}$ kinetics has not been explored in detail. In previously untrained but healthy individuals, the speeding of ${\dot{\text{V}}\text{O}}_{{2}}$ kinetics occurs acutely (i.e., within the first 1–4 training sessions) following very short interventions (Phillips et al. 1995; McKay et al. 2009; Murias et al. 2016; McLay et al. 2017; Iannetta et al. 2022a) or within the first weeks of short-term (i.e. < 12 weeks) endurance training programs (Phillips et al. 1995; Murias et al. 2010, 2011a); however, the extent to which the nature of the training stimulus impacts the speeding of ${\dot{\text{V}}\text{O}}_{{2}}$ kinetics is unclear. Current lines of evidence indicate that the metabolic disturbance elicited by the exercise intensity is a key component determining cardiovascular adaptations to endurance training, at least when considering changes in ${\dot{\text{V}}\text{O}}_{{{\text{2max}}}}$ or maximal metabolic steady state (MMSS) following an endurance exercise training program (Hawley et al. 2014; Weatherwax et al. 2019; Inglis et al. 2024). However, the effects of domain-specific exercise intensity training on ${\dot{\text{V}}\text{O}}_{{2}}$ kinetics have not been evaluated.

Previously, it has been shown that there were no differences in the speeding of ${\dot{\text{V}}\text{O}}_{{2}}$ kinetics when comparing high-intensity interval training (HIIT) and continuous training (Berger et al. 2006; McKay et al. 2009), suggesting that the faster ${\dot{\text{V}}\text{O}}_{{2}}$ kinetics is not intensity-dependent. Similarly, two short-term continuous exercise training programs of different intensities (i.e., 50% and 70% of ${\dot{\text{V}}\text{O}}_{{{\text{2max}}}}$) elicited similar speeding of the ${\dot{\text{V}}\text{O}}_{{2}}$ kinetics response (Murias et al. 2016). However, the intensity of the continuous training was prescribed as a percentage of ${\dot{\text{V}}\text{O}}_{{{\text{2max}}}}$, a method that has been demonstrated to lack accuracy in ensuring uniform metabolic disturbance across individuals (Scharhag-Rosenberger et al. 2009; Iannetta et al. 2020; Meyler et al. 2023). Furthermore, the aforementioned exercise interventions were work-matched in only one of the studies (Berger et al. 2006), which does not allow for the isolation of the role of intensity.

Although ${\dot{\text{V}}\text{O}}_{{2}}$ kinetics is proposed to be a determinant of performance (Jones and Burnley 2009; Rossiter 2011; Goulding et al. 2021), based partly on the association between ${\dot{\text{V}}\text{O}}_{{2}}$ kinetics and performance related outcomes such as critical power (Rossiter 2011; Goulding et al. 2021), data directly linking changes in ${\dot{\text{V}}\text{O}}_{{2}}$ kinetics with changes in performance outcomes are lacking. For example, Rossiter (2011) showed a negative correlation between critical power and $\tau {\dot{\text{V}}\text{O}}_{{2}}$ across different populations (i.e., young healthy trained and untrained, healthy older, and patients with chronic obstructive pulmonary disease), suggesting that these variables are associated. However, these data came from cross-sectional studies, and interventional studies that can determine whether a speeding ${\dot{\text{V}}\text{O}}_{{2}}$ kinetics directly improves performance are needed.

Thus, the main goals of this study were to: (i) examine the effect of training at different exercise intensities on the speeding of ${\dot{\text{V}}\text{O}}_{{2}}$ kinetics, following 3 and 6 weeks of exercise training using an intensity domain-based approach; and (ii) evaluate the relationships between changes in ${\dot{\text{V}}\text{O}}_{{2}}$ kinetics with concomitant changes in indices of exercise performance as measured by ${\dot{\text{V}}\text{O}}_{{{\text{2max}}}}$, estimated lactate threshold (θ_LT_), MMSS, and ramp-incremental peak power output (PPO). As a secondary goal, this study evaluated the relationships between $\Delta \tau {\dot{\text{V}}\text{O}}_{{2}}$ and Δ near-infrared spectroscopy (NIRS) derived oxidative capacity (OxCap) to explore potential mechanistic links for the speeding of the ${\dot{\text{V}}\text{O}}_{{2}}$ response. Based on previous research demonstrating rapid and similar speeding of ${\dot{\text{V}}\text{O}}_{{2}}$ kinetics following exercise training of different (but not precisely controlled) intensities, we hypothesized that a speeding of ${\dot{\text{V}}\text{O}}_{{2}}$ kinetics would be similar regardless of exercise intensity. Additionally, in connection with the idea of similar speeding of the ${\dot{\text{V}}\text{O}}_{{2}}$ kinetics across all interventions despite intensity-dependent changes in maximal outcomes, we hypothesized that the speeding of ${\dot{\text{V}}\text{O}}_{{2}}$ kinetics would not be correlated with changes in performance outcomes. Finally, we hypothesized that potential changes in OxCap would not be significantly correlated with changes in $\tau {\dot{\text{V}}\text{O}}_{{2}}$.

## Methods

### Participants

The data presented in this study are part of the MEGA training study of which partial data have been presented (Inglis et al. 2024). As previously indicated (Inglis et al. 2024), ninety-nine young healthy females (n = 49) and males (n = 50) volunteered and provided written informed consent to participate after completing the physical activity readiness questionnaire (PAR-Q +) and being cleared for exercise by a certified exercise physiologist. Due to illness/injury (n = 5), COVID-19 related precautions (n = 4), or other personal reasons (n = 6), fifteen of these participants were unable to complete the study. Thus, eighty-four participants (42 F, 42 M) ranging from sedentary to recreationally active volunteered to participate in the study. Stratified randomization based on sex was used to equally assign participants to one of five cycling exercise intervention groups (n = 70) or control (CON) (n = 14). ${\dot{\text{V}}\text{O}}_{{2}}$ kinetics data for three participants were not collected due to technical issues and thus their data for the other measured variables are not presented. The sample size for the MEGA project was calculated considering the main outcome variable (i.e., maximal ${\dot{\text{V}}\text{O}}_{{2}}$.). However, the post analysis statistical power for $\tau {\dot{\text{V}}\text{O}}_{{2}}$ in this study indicated values greater than 0.8 for the main effects of time and group. All procedures included in this study were approved by the Conjoint Health Research Ethics Board at the University of Calgary (REB19-0452) and complied with the principles in latest version of the Declaration of Helsinki.

### Equipment

A metabolic cart and mixing chamber (Quark CPET; COSMED, Rome, Italy) were used to measure gas exchange and ventilatory variables continuously during maximal and MMSS testing sessions. A breath-by-breath setup was used for the submaximal protocol designed to evaluate ${\dot{\text{V}}\text{O}}_{{2}}$ kinetics. Prior to each testing session, gas and flow calibrations were performed according to manufacturer recommendations. Heart rate was also monitored continuously during all testing and training sessions (Garmin, Chicago, IL). Blood lactate concentration ([La^−^]) was measured from capillary blood samples obtained from a prick of the finger (Biosen C-Line, EKF Diagnostics, Barleben, Germany).

All maximal, MMSS and training sessions (apart from sprint interval training (SIT)) were performed using the Tacx NEO Smart Trainers (Garmin, Chicago, IL, USA) with custom, adjustable bike frames. A tablet with the Golden Cheetah software (version 3.4; https://www.goldencheetah.org/) was used to control the protocols. Participants were individually assigned to a training station that was kept consistent throughout the study. The ${\dot{\text{V}}\text{O}}_{{2}}$ kinetics protocol and SIT was performed on an electromagnetically braked cycle ergometer (Velotron Dynafit Pro, Racer Mate, Seattle, WA, USA) using the Velotron CS and Wingate software, respectively. For all training and testing sessions with the NEO system, participants were asked to maintain their preferred cadence between 70 and 95 rpm. This range was selected to accommodate the individual preferences of participants with limited cycling experience to make sure that they did not cycle at a cadence that was not comfortable for them. The majority of the participants had a preferred cadence between 75 and 85 rpm. Once a preferred cadence was established this was kept consistent throughout the study.

### Experimental timeline and protocols

Participation in the study lasted approximately 11.5 weeks, with testing phases occurring before (PRE), between (MID), and immediately following (POST) exercise training. The intervention was composed of two, 3-week training phases (6 weeks total). Each testing phase consisted of one ramp incremental maximal session, 2–3 sessions to determine MMSS, and one fasted submaximal testing session. Time of day for the testing sessions was kept consistent (± 1 h) for each participant at PRE, MID and POST. A minimum of 24 h separated the maximal session from the first MMSS session, 48–72 h separated subsequent MMSS trials, and the fasted submaximal session was performed following a minimum of 48 h without testing/training. The CON group performed PRE and POST testing.

Maximal session. To measure ${\dot{\text{V}}\text{O}}_{{{\text{2max}}}}$, HR_max_, PPO, θ_LT_, and the respiratory compensation point (RCP), participants performed a cycling ramp incremental test (Females: 20 W·min^−1^, Males: 25 W·min^−1^) to task failure. Prior to the ramp, participants performed 4 min of cycling at 30 W followed by 8 min of cycling in the moderate intensity domain (60–80 W individual dependent). Then, following 3 min of rest, participants performed a 4-min bout of cycling at 30 W before the ramp portion of the test began. Participants cycled until task failure, defined as the point at which there was a drop in cadence below 65 rpm for more than 5 consecutive seconds or participants could not continue to cycle, despite strong verbal encouragement. Then, after a brief recovery period (~ 7–10 min), participants performed a constant work rate trial that consisted of a 2-min baseline at 30 W followed by square wave step transition to ~ 80–85% of PPO performed to task failure. The purpose of this trial was to induce maximal responses for a separate aim of the MEGA study. Immediately (within 30 s) following both the ramp-incremental and the constant work rate trial, blood lactate ([La^−^]) samples were taken.

MMSS Sessions. Participants completed 2–3 constant PO exercise trials to determine the power output (PO) associated with MMSS. Each trial began with 4 min of cycling at 30 W, followed by an instantaneous increase to a pre-determined PO for 30 min during which [La^−^] was measured at 5 min intervals and ${\dot{\text{V}}\text{O}}_{{2}}$ was measured continuously. The PO for the initial trial was determined using a predictive equation based on measured RCP (Iannetta et al. 2018). For subsequent trials, and to obtain measures of MMSS PO with a small margin of error, the PO was increased or decreased by 5% of PO based on the [La^−^] and ${\dot{\text{V}}\text{O}}_{{2}}$ responses. Blood [La^−^] samples were obtained at baseline and at 5-min intervals thereafter in duplicate or triplicate (15th and 30th min), and each timepoint was represented by the average of the two available samples or the average of the two closest samples when taken in triplicate. As previously defined (Iannetta et al. 2022b), MMSS was determined to be the highest PO at which stable [La^−^] and V̇O_2_ responses were achieved between the 15th and 30th min of exercise.

### Fasted submaximal protocols

${\dot{\text{V}}\text{O}}_{{2}}$ kinetics. The ${\dot{\text{V}}\text{O}}_{{2}}$ kinetics protocol consisted of three cycling moderate step transitions (Spencer et al. 2011) from a 20-W baseline period (6 min) to 80–85% of the PO associated with the θ_LT_ (6 min) at PRE using a square wave transition. The PO was kept consistent at PRE, MID and POST for each participant. Throughout the protocol, participants were asked to maintain a steady cadence in the range of 65–75 rpm.

NIRS derived Oxidative Capacity. A NIRS probe (PortaMon, Artinis Medical Systems, Elst, The Netherlands) was used to provide an indirect measure of mitochondrial oxidative capacity as previously described (Hamaoka et al. 1996; Motobe et al. 2004; Rasica et al. 2024). Briefly, the NIRS probe was placed on the lower third of the vastus lateralis (VL) and covered with an elastic bandage to prevent movement of the probe and the intrusion of external light. A cuff connected to a pneumatic automatic rapid inflation system (Hokanson E20, Bellevue, WA, USA) was placed on the proximal portion of the right thigh (above the VL NIRS probe) to occlude blood flow to the leg. Ankle weights (3.5 kg for females and 4.5 kg for males) were placed on the ankle of the right leg. First, participants performed 20 repetitions of knee extension in a seated position at 0.5 Hz (1 s extension, 1 s flexion). Immediately after, the cuff was inflated to 300 mmHg for 5 s and subsequently deflated for 10 s and this inflation-deflation procedure was repeated 20 times over a 5-min period. Following 2-min of rest, once the NIRS signals had stabilized, a second trial was performed. NIRS variables were recorded continuously (10 Hz) throughout the repeated occlusion protocol.

### Endurance exercise training prescription

Exercise training was separated into two 3-week phases, each consisting of nine sessions (three sessions per week) for a total of 18 sessions at the end of the program. Exercise training sessions were completed Monday through Saturday with ≥ 24 h between sessions and at least one 48 h rest period within each week (i.e., participants were not allowed to exercise on three consecutive days). All training sessions were supervised and adherence across all intervention groups was 98 ± 5%, as previously described (Inglis et al. 2024). Detailed information on reasons for the selected intensities and durations for the five exercise interventions have been presented elsewhere (Inglis et al. 2024). Briefly, the groups performed cycling at a constant-PO in the (i) moderate-intensity domain (MOD) (50-min of cycling at 90% of the PO at θ_LT_); (ii) lower boundary of the heavy-intensity domain (HVY1) (~ 41-min at 110% of the PO at θ_LT_); (iii) upper boundary of the heavy-intensity domain (HVY2) (~ 30-min at 100% of the PO at MMSS); or interval training in the form of (iv) high-intensity interval training (HIIT) in the severe-intensity domain (5 to 6 intervals with a 4:3-min work:rest ratio); and (v) SIT in the extreme-intensity domain (up to 6, 30 s maximal-effort sprints against a fixed resistance (0.06 to 0.075 kg-kg^−1^ body mass)). Participants in the MOD, HVY1, HVY2, and HIIT groups performed a standardized 2-min warmup at 55% of θ_LT_ whereas the SIT group performed a 10-min warmup at 50 W with three 3-s practice sprints interspaced within the later half. SIT participants started with three sprints per session, with the number of sprints per session progressively increasing by a minimum of one sprint per week until six sprints were performed by week four. The total work performed in HVY1, HVY2, and HIIT were individually work-matched to the total work of each participants’ MOD equivalent (i.e., 50-min of cycling at 90% of the PO at θ_LT_). The training intensities in phases 1 and 2 were based on the results of testing at PRE and MID, respectively (i.e., the training load was adjusted following MID to ensure adequate classification within the domain-specific regions).

### Data analysis

Raw ventilatory and gas-exchange data from the ramp incremental test were independently evaluated by two experts to identify the ${\dot{\text{V}}\text{O}}_{{2}}$ corresponding to the θ_LT_ and RCP. In case of a disagreement of > 100 mL/min, both experts re-evaluated the profile together until an agreement was reached. The θ_LT_ and RCP were established as previously detailed (Beaver et al. 1986; Gaesser and Wilson 1988; Whipp et al. 1989; Keir et al. 2022).

${\dot{\text{V}}\text{O}}_{{2}}$ data were filtered by removing aberrant points that fell 3 standard deviations from the local mean prior to being linearly interpolated on a second-by-second basis. The moderate step transition performed prior to the ramp-incremental portion of the test was used to calculate the ${\dot{\text{V}}\text{O}}_{{2}}$ mean response time as previously described (Iannetta et al. 2019). Briefly, a linear fit was applied to the ramp-incremental test V̇O_2_ data from the ramp onset to the established θ_LT_. The ${\dot{\text{V}}\text{O}}_{{2}}$ of the moderate step transition (average of the last 2-min) was then superimposed on the ${\dot{\text{V}}\text{O}}_{{2}}$ *vs* PO relationship from the ramp. Then, the difference in PO corresponding to the abscissa identified during the ramp linear fit *versus* that of the moderate-step transition was converted to time and then used to align the ramp-incremental ${\dot{\text{V}}\text{O}}_{{2}}$ with the corresponding PO, allowing for the identification of PO at the θ_LT_. The highest 20-s rolling average from either the ramp incremental test or the subsequent constant work rate trial was defined as ${\dot{\text{V}}\text{O}}_{{{\text{2max}}}}$.

${\dot{\text{V}}\text{O}}_{{2}}$ kinetics. As previously described (Keir et al. 2014), the ${\dot{\text{V}}\text{O}}_{{2}}$ data for each step transition were cleaned, time aligned, and interpolated before being ensemble-averaged into a single time-averaged response. Then, each individual ensemble-averaged profile was time-averaged into 5 s bins (Keir et al. 2014) and fit using the following equation:

$${\dot{\text{V}}\text{O}}_{{2}} \left( {\text{t}} \right) \, = {\dot{\text{V}}\text{O}}_{{{\text{2bsln}}}} + {\dot{\text{V}}\text{O}}_{{{\text{2AMP }} \cdot }} \left( {{1 } - {\text{ e}}^{{ - \, \left( {{\text{t}} - {\text{TD}}} \right)/\tau }} } \right)$$

where ${\dot{\text{V}}\text{O}}_{{2}} \left( {\text{t}} \right)$ represents the ${\dot{\text{V}}\text{O}}_{{2}}$ at any given time (t) during the transition, ${\dot{\text{V}}\text{O}}_{{{\text{2bsln}}}}$ is the steady-state baseline value of ${\dot{\text{V}}\text{O}}_{{2}}$ before the moderate step-transition, ${\dot{\text{V}}\text{O}}_{{{\text{2AMP}}}}$ is the amplitude of the increase in ${\dot{\text{V}}\text{O}}_{{2}}$ above ${\dot{\text{V}}\text{O}}_{{{\text{2bsln}}}}$, TD is the time delay of the response, and τ is the time constant of the response (defined as the time required to attain 63% of the steady-state amplitude). To account for the phase I (i.e., the cardiodynamic phase) of the ${\dot{\text{V}}\text{O}}_{{2}}$ response, the first 20 s of the ensemble-averaged ${\dot{\text{V}}\text{O}}_{{2}}$ profile were not included in the fitting window of the phase II ${\dot{\text{V}}\text{O}}_{{2}}$ (i.e., the primary component reflecting the adjustment of muscle ${\dot{\text{V}}\text{O}}_{{2}}$) across all participants as previously recommended (Murias et al. 2011b). Data were modelled from the beginning of phase II up to 240 s of the step-transition, after ensuring that steady-state ${\dot{\text{V}}\text{O}}_{{2}}$ had been attained within this time window. The parameter estimates were computed by least squares non-linear regression using the whippr open-source R package, with the best fit defined by minimization of the residual sum of squares and minimal variation around the Y-axis (${\dot{\text{V}}\text{O}}_{{2}}$ = 0). The 95% confidence interval for the estimated τ was determined after preliminary fit of the data with ${\dot{\text{V}}\text{O}}_{{{\text{2bsln}}}}$, ${\dot{\text{V}}\text{O}}_{{{\text{2AMP}}}}$, and TD constrained to the best fit values and the τ allowed to vary.

Oxidative Capacity. ${\dot{\text{V}}\text{O}}_{{2}}$ at the level of the muscle (${\dot{\text{V}}\text{O}}_{{2}} {\text{m}}$) was estimated by calculating the slope of saturation (StO_2_) over a 3-s span of data, excluding the data points from the first and last second of each occlusion period to avoid any potential influence from cuff inflation or release.

${\dot{\text{V}}\text{O}}_{{2}} {\text{m}}$ values were respectively fit by the following monoexponential function:

$${\text{Y}}\left( {\text{t}} \right) = {\text{y}}_{{{\text{END}}}} - {\text{A}} \times {\text{e}}^{{ - \frac{1}{\tau }}}$$

where ${\text{Y}}\left( {\text{t}} \right)$ = ${\dot{\text{V}}\text{O}}_{{2}} {\text{m}}$ at a given time (t); ${\text{y}}_{\text{END}}$ = ${\dot{\text{V}}\text{O}}_{{2}} {\text{m}}$ immediately after the cessation of the exercise; A = amplitude of the response; τ = exponential recovery rate constant (τ, τ = $\frac{1}{\text{k}}$).

As previously indicated (Beever et al. 2020), prior to fitting, the data were visually inspected to remove invalid values or outliers possibly caused by partial occlusions or disturbances in the NIRS signal. After identifying the start of the monoexponential decay curve, data points preceding this point were not considered for the curve fitting. Points within the plateau were only removed if they dissociated enough from the curve or plateau to suggest that the point was invalid (Beever et al. 2020). A single value of τ (τOxCap) was reported for each participant, which was determined by taking the average τ obtained from the two trials (Rasica et al. 2024). Due to technical issues (i.e., adipose tissue thickness causing NIRS signal disturbance), the OxCap analysis was completed in 70 of the 84 participants (CON = 10, MOD = 13, HVY1 = 12, HVY2 = 13, HIIT = 11, SIT = 11). The r^2^ of the fits for each group ranged from 0.92–0.96, indicating a high quality fit.

### Statistics

A one-way ANOVA was used to compare baseline group characteristics groups. A paired-samples t-test was used to evaluate changes within the CON group from PRE to POST. To compare the effect of time (PRE, MID, and POST) and group on the variables of interest in the intervention group, a mixed model ANOVA was performed. Tukey’s post-hoc tests were used to confirm significance among multiple comparisons. Effect sizes are reported as partial eta-squared (partial η^2^), where values of 0.01, 0.06 and 0.14 correspond to small medium and large effects, respectively (Lakens 2013). A Pearson product moment correlation was used to determine the strength of relationship between changes in $\tau {\dot{\text{V}}\text{O}}_{{2}}$ and performance variables (${\dot{\text{V}}\text{O}}_{{{\text{2max}}}}$, θ_LT,_ MMSS and PPO). The strength of association for the correlation values was defined as strong (|r|> 0.5), (medium 0.3 >|r|), or weak (0.3 >|r|> 0.1). Statistical significance was set at p < 0.05. Statistical analyses were performed using SPSS Statistics v. 26.0 (SPSS; IBM, Chicago, IL).

## Results

At PRE, there were no between group differences for age (p = 0.91), height (p = 0.75), body mass (p = 0.90), ${\dot{\text{V}}\text{O}}_{{{\text{2max}}}}$ (p = 0.87), and PPO (p = 0.87) (Table 1). Additionally, there were no between-group differences in the PO values at θ_LT_ (p = 0.62) and MMSS (p = 0.75) (Table 1).

**Table 1:** Participant characteristics and physiological variables at PRE, MID and POST for the intervention groups

	MOD (n = 14)			HVY1 (n = 14)			HVY2 (n = 13)			HIIT (n = 13)			SIT (n = 14)		
	PRE	MID	MID	PRE	MID	POST	PRE	MID	POST	PRE	MID	POST	PRE	MID	POST
Age															
Years	25 ± 5			27 ± 5			26 ± 5			26 ± 6			28 ± 6		
Height															
Cm	167 ± 8			170 ± 8			169 ± 7			170 ± 11			172 ± 11		
Body mass															
Kg	66.4 ± 11.9	66.1 ± 1.6	66.1 ± 11.3	71.7 ± 13.1	71.5 ± 12.3	71.5 ± 11.9	68.5 ± 8.5	68.8 ± 8.3	68.6 ± 8.4	72.2 ± 17.3	72.3 ± 16.8	72.0 ± 15.7	73.9 ± 10.7	73.2 ± 10.7	72.5 ± 11.1
$${\dot{\mathbf{V}}\mathbf{O}}_{{{\mathbf{2max}}}}$$															
mL·kg^−1^·min^−1^	41.1 ± 5.7	42.1 ± 5.7	42.9 ± 5.2^ǂ^	40.1 ± 5.5	42.8 ± 6.0^ǂ^	43.4 ± 5.4^ǂ^	40.9 ± 5.0	45.3 ± 4.7^ǂ^	46.4 ± 4.5^ǂ†^	40.2 ± 6.4	43.4 ± 7.1^ǂ^	46.2 ± 7.1^ǂ**^	40.9 ± 6.6	43.7 ± 7.1^ǂ^	45.6 ± 6.9^ǂ**^
PPO															
Watts	221 ± 49	230 ± 47^ǂ^	238 ± 50^ǂ**^	231 ± 51	251 ± 54^ǂ^	257 ± 56^ǂ**^	228 ± 42	257 ± 41^ǂ^	265 ± 41^ǂ**^	218 ± 55	255 ± 63^ǂ^	269 ± 61^ǂ**^	247 ± 52	258 ± 54^ǂ^	266 ± 55^ǂ**^
θ_LT_															
Watts	87 ± 26	97 ± 22	103 ± 28	93 ± 22	114 ± 30	119 ± 27^ǂ^	104 ± 32	122 ± 28	132 ± 28^ǂ**^	97 ± 30	109 ± 36^ǂ^	121 ± 32^ǂ**^	95 ± 24	103 ± 25	120 ± 26^ǂ**^
MMSS															
Watts	125 ± 31	131 ± 34^ǂ^	136 ± 33^ǂ**^	133 ± 33	144 ± 36^ǂ^	152 ± 36^ǂ**^	135 ± 36	150 ± 35^ǂ^	157 ± 33^ǂ**^	127 ± 45	144 ± 46^ǂ^	156 ± 47^ǂ**^	137 ± 36	147 ± 39^ǂ^	153 ± 39^ǂ**^

Values are mean ± standard deviation; ^ǂ^Significantly different from PRE, **Significantly different from MID

**Fig. 1:**
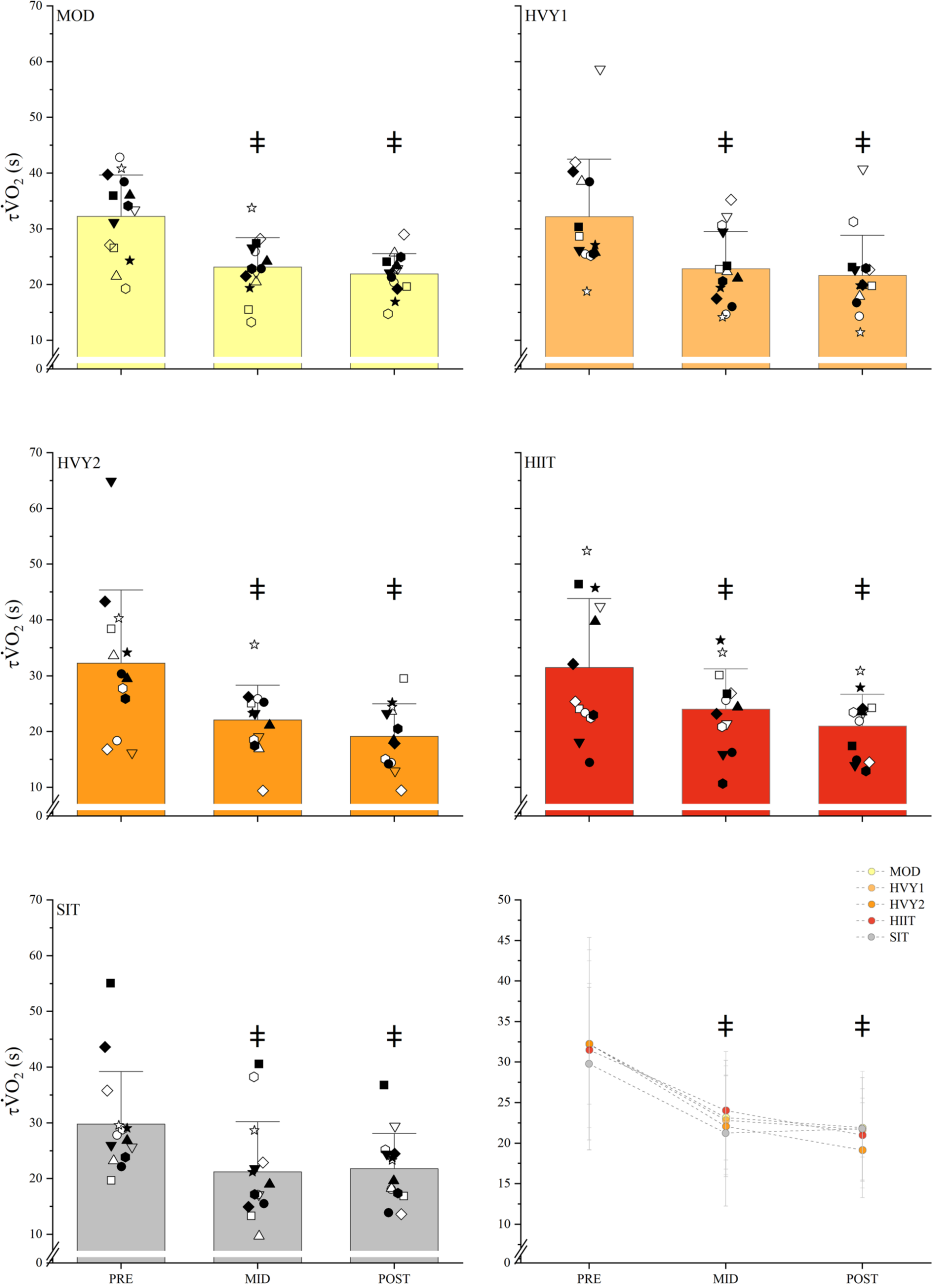
Group, individual and average $\tau {\dot{\text{V}}\text{O}}_{{2}}$ values for the intervention groups at PRE, MID, and POST. ^ǂ^Significantly different from PRE

${\dot{\text{V}}\text{O}}_{{2}}$ Kinetics. For $\tau {\dot{\text{V}}\text{O}}_{{2}}$, there was no significant interaction effect (F(6.30, 99.30) = (0.46, p = 0.843, η^2^ = 0.03, 0.00–0.03)) but there was a significant time effect F(F(1.58, 99.3) = (69.29, p < 0.001, η^2^ = 0.52, 0.41–0.60)), with differences between PRE and MID (p < 0.001) and PRE and POST (p < 0.001) and no difference between MID and POST (p = 0.067) (Table 2; Fig. 1). For baseline V̇O_2_, there was no significant interaction effect (F(6.85, 107.89) = (0.52, p = 0.816, η^2^ = 0.03, 0.00–0.03)) but a time effect (F(1.71, 107.89) = (12.91, p < 0.001, η^2^ = 0.17, 0.07–0.27)) between PRE and MID (p = 0.014) and PRE and POST (p < 0.001) but no difference between MID and POST (p = 0.118) (Table 2). Steady-state V̇O_2_ showed no significant interaction effect (F(6.37, 100.25) = (0.68, p = 0.678, η^2^ = 0.04, 0.00–0.06)) but a significant time effect (F(1.59, 100.25) = (7.02, p = 0.003, η^2^ = 0.10, 0.02–0.19)) with differences between PRE and POST (p = 0.004) but no differences between PRE and MID (p = 0.071) nor MID and POST (p = 0.557). There was no interaction effect for ${\dot{\text{V}}\text{O}}_{{2}}$ amplitude (F(6.34, 99.98) = (0.84, p = 0.549, η^2^ = 0.05, 0.00–0.07)) and no time effect (F(1.59, 99.98) = (0.30, p = 0.694, η^2^ = 0.01, 0.00–0.04)). The ${\dot{\text{V}}\text{O}}_{{2}}$ gain demonstrated no interaction effect (F(7.08, 111.57) = (1.06, p = 0.394, η^2^ = 0.06, 0.00–0.09)), and no time effect (F(1.771, 111.57) = (0.21, p = 0.784, η^2^ = 0.00, 0.00–0.03)) with differences between HIIT and SIT.

**Table 2:** Mean and standard deviation values for the control group

	CONTROL (n = 13)	
	PRE	POST
Age		
Years	27 ± 5	
Height		
Cm	170 ± 7	
Body mass		
Kg	69.0 ± 12.2	69.3 ± 12.1
$${\dot{\text{V}}\text{O}}_{{{\text{2max}}}}$$		
mL·kg^−1^·min^−1^	42.9 ± 6.2	43.0 ± 5.9
PPO		
W	240 ± 52	241 ± 53
θ_LT_		
Watts	96 ± 18	99 ± 22
MMSS		
W	141 ± 35	142 ± 35
Baseline		
L·min^−1^	0.77 ± 0.12	0.77 ± 0.13
Steady state		
L·min^−1^	1.31 ± 0.25	1.31 ± 0.26
Amplitude		
L·min^−1^	0.54 ± 0.16	0.54 ± 0.16
Gain		
mL·W^−1^	9.8 ± 0.8	9.7 ± 0.6
τ		
s	27.2 ± 7.8	25.6 ± 11.9
95%CI		
s	5.7 ± 1.8	5.4 ± 1.7
Time delay		
s	12.9 ± 3.0	11.4 ± 5.1

Oxidative Capacity. Measures of oxidative capacity at PRE, MID and POST were 23.7 ± 7.6 s, 20.8 ± 5.6 s, 21.5 ± 7.0 s (MOD, n = 13), 28.0 ± 9.0 s, 21.1 ± 7.7 s, 19.8 ± 8.9 s (HVY1, n = 11), 26.1 ± 6.0 s, 22.5 ± 8.8 s, 20.8 ± 4.7 s (HVY2, n = 12), 29.6 ± 7.0 s, 23.0 ± 5.3 s, 20.8 ± 3.7 s (HIIT, n = 10), and 23.2 ± 7.3 s, 23.2 ± 6.0 s, 23.0 ± 6.3 s (SIT, n = 11), respectively. There was no significant interaction effect (F(8,104) = (1.52, p = 0.158, η^2^ = 0.11, 0.00–0.14)), but there was a significant time effect (F(2,104) = (13.64, p < 0.001, η^2^ = 0.21, 0.09–0.31)) for τOxCap, with differences found from PRE to MID (p = 0.001) and PRE to POST (p < 0.001) but not from MID to POST (p = 0.963).

${\dot{\text{V}}\text{O}}_{{{\text{2max}}}}$, Thresholds, and PPO. For CON, which was assessed only at PRE and POST, there were no statistical differences in any of the evaluated outcomes (p < 0.05) (Table 3). For the intervention groups, a significant (time x intensity) interaction was identified for ${\dot{\text{V}}\text{O}}_{{{\text{2max}}}}$ (F(6.72, 105.85) = (5.03, p < 0.001, η^2^ = 0.24, 0.11–0.34)). HVY2, HIIT and SIT demonstrated increases in ${\dot{\text{V}}\text{O}}_{{{\text{2max}}}}$ from PRE to MID and POST, as well as MID to POST (p < 0.05) (Table 1). HVY1 demonstrated an increase PRE to MID and PRE to POST (p < 0.0.001) but not MID to POST (p = 0.445). MOD resulted in a greater ${\dot{\text{V}}\text{O}}_{{{\text{2max}}}}$ from PRE to POST (p = 0.029) but not at PRE to MID (p = 0.367) or MID to POST (p = 0.190). PO at θ_LT_ showed no significant interaction effect (F(7.81, 122.95) = (1.82, p = 0.081, η^2^ = 0.10, 0.00–0.14)), but there was a significant time effect (F(1.95,122.95) = (101.55, p < 0.001, η^2^ = 0.62, 0.53–0.68)), with increases at each time point. All intervention groups demonstrated increases from PRE to MID, PRE to POST and MID to POST for PO at MMSS (F(6.27,98.77) = (5.50, p < 0.001, η^2^ = 0.26, 0.10–0.33)) and PPO (interaction (F(5.87, 92.4) = (11.55, p < 0.001, η^2^ = 0.42, 0.26–0.50)). PRE to POST changes for these indices of performance have been previously presented, but not in connection to the V̇O_2_ responses (Inglis et al. 2024).

**Table 3:** ${\dot{\text{V}}\text{O}}_{{2}}$ kinetics variables at PRE, MID and POST for the intervention groups

	MOD (n = 14)			HVY1 (n = 14)			HVY2 (n = 13)			HIIT (n = 13)			SIT (n = 14)		
	PRE	MID	POST	PRE	MID	POST	PRE	MID	POST	PRE	MID	POST	PRE	MID	POST
Baseline ${\dot{\text{V}}\text{O}}_{{2}}$															
L·min^−1^	0.80 ± 0.13	0.77 ± 0.10^ǂ^	0.73 ± 0.08^ǂ^	0.82 ± 0.12	0.76 ± 0.10^ǂ^	0.77 ± 0.08^ǂ^	0.82 ± 0.10	0.79 ± 0.08^ǂ^	0.78 ± 0.05^ǂ^	0.87 ± 0.18	0.80 ± 0.12^ǂ^	0.77 ± 0.12^ǂ^	0.83 ± 0.16	0.80 ± 0.11^ǂ^	0.78 ± 0.08^ǂ^
Steady State ${\dot{\text{V}}\text{O}}_{{2}}$															
L·min^−1^	1.33 ± 0.31	1.32 ± 0.28	1.25 ± 0.28^ǂ^	1.36 ± 0.23	1.30 ± 0.21	1.33 ± 0.20^ǂ^	1.43 ± 0.26	1.40 ± 0.26	1.37 ± 0.23^ǂ^	1.52 ± 0.43	1.45 ± 0.31	1.42 ± 0.30^ǂ^	1.39 ± 0.30	1.33 ± 0.19	1.34 ± 0.19^ǂ^
Amplitude															
L·min^−1^	0.54 ± 0.21	0.55 ± 0.22	0.53 ± 0.23	0.55 ± 0.18	0.54 ± 0.19	0.57 ± 0.19	0.62 ± 0.25	0.62 ± 0.22	0.60 ± 0.20	0.67 ± 0.28	0.65 ± 0.22	0.65 ± 0.21	0.57 ± 0.19	0.54 ± 0.16	0.57 ± 0.16
Gain															
mL·W^−1^	10.1 ± 1.5	10.4 ± 1.2	9.8 ± 1.0	9.9 ± 0.9	9.5 ± 1.0	10.1 ± 1.0	9.7 ± 1.3	9.8 ± 0.9	9.6 ± 1.1	10.4 ± 1.4^#^	10.3 ± 0.8^#^	10.3 ± 0.9^#^	9.7 ± 1.4	9.1 ± 0.8	9.6 ± 0.6
τ															
s	32.2 ± 7.4	23.1 ± 5.2^ǂ^	21.9 ± 3.6^ǂ^	32.2 ± 10.3	22.8 ± 6.7^ǂ^	21.6 ± 7.2^ǂ^	32.3 ± 13.1	22.1 ± 6.2^ǂ^	19.1 ± 5.8^ǂ^	31.5 ± 12.3	24.0 ± 7.2^ǂ^	21.8 ± 6.3^ǂ^	29.8 ± 9.4	21.2 ± 9.0^ǂ^	21.7 ± 6.3^ǂ^
95%CI															
s	6.3 ± 2.6	6.2 ± 3.0	5.3 ± 2.4	5.7 ± 2.3	6.0 ± 4.9	5.1 ± 2.2	6.0 ± 2.8	5.1 ± 2.7	4.6 ± 2.5	5.0 ± 1.4	4.8 ± 1.8	4.0 ± 1.3	5.9 ± 3.3	5.5 ± 3.4	5.5 ± 3.8
Time delay															
s	11.9 ± 3.7	14.0 ± 2.8	14.8 ± 3.4	11.3 ± 5.7	13.4 ± 2.7	12.5 ± 5.6	11.4 ± 4.2	14.0 ± 2.9	14.8 ± 2.9	10.7 ± 5.8	12.4 ± 4.8	12.9 ± 4.3	9.9 ± 4.6	14.4 ± 2.0	12.2 ± 4.0

Values are mean ± standard deviation. ^ǂ^Significantly different from PRE, ^#^significantly different from SIT

**Fig. 2:**
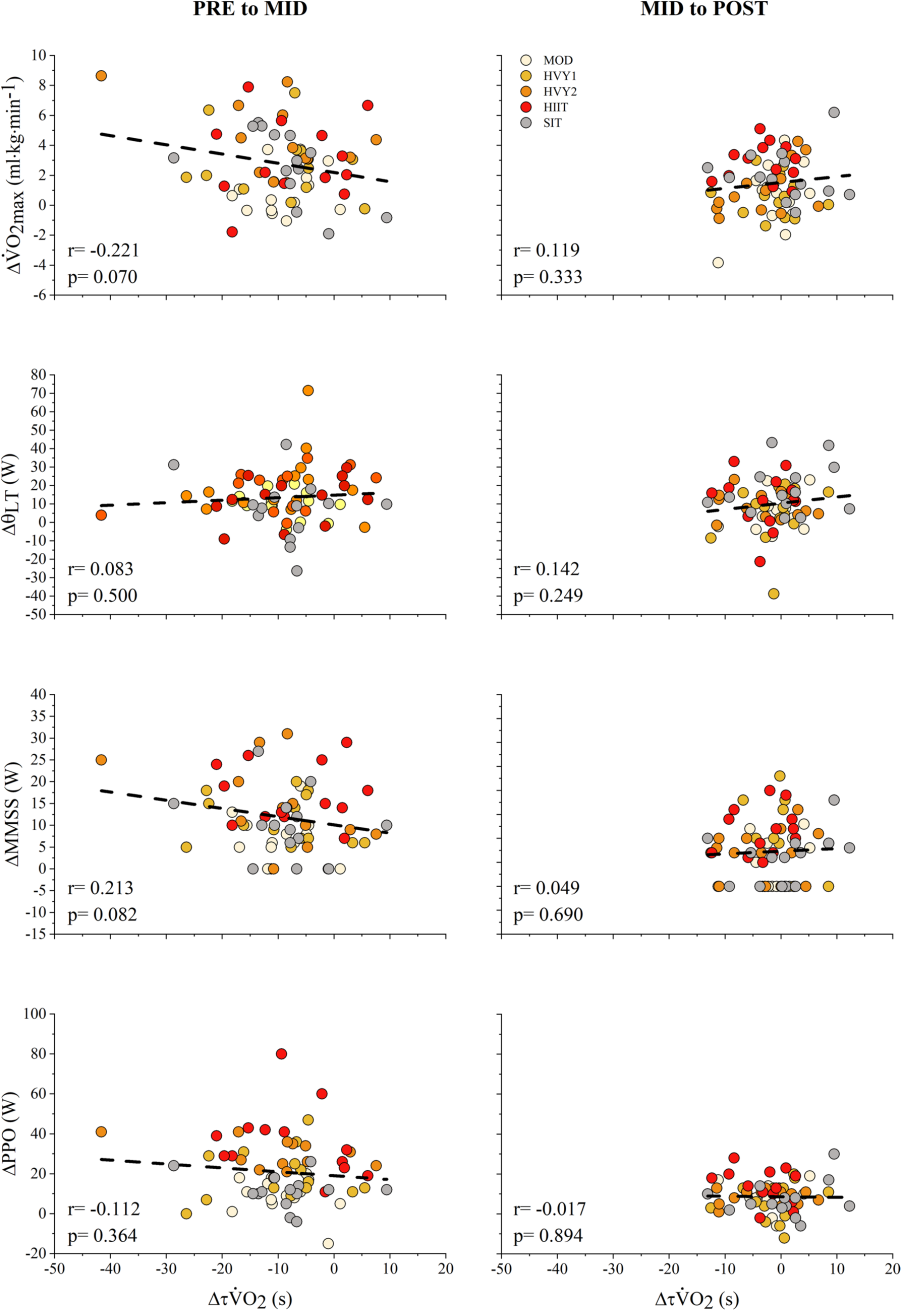
Correlations between $\Delta \tau {\dot{\text{V}}\text{O}}_{{2}}$ and $\Delta {\dot{\text{V}}\text{O}}_{{{\text{2max}}}}$, Δθ_LT_, ΔMMSS, and ΔPPO at PRE to MID and MID to POST

Correlations with Indices of Performance. There were no significant correlations between $\Delta \tau {\dot{\text{V}}\text{O}}_{{2}}$ and $\Delta {\dot{\text{V}}\text{O}}_{{{\text{2max}}}}$, ΔPPO, Δθ_LT_ and ΔMMSS from PRE to MID and MID to POST (Fig. 2). Additionally, no significant correlations were found between $\Delta \tau {\dot{\text{V}}\text{O}}_{{2}}$ and ΔτOxCap from PRE to MID (r = 0.009, p = 0.948) and MID to POST (r = 0.133, p = 0.325).

## Discussion

We investigated changes in ${\dot{\text{V}}\text{O}}_{{2}}$ kinetics in response to intensity domain-specific endurance exercise training in young and previously untrained females and males and evaluated the relationship between changes in $\tau {\dot{\text{V}}\text{O}}_{{2}}$ and those associated with performance indices such as ${\dot{\text{V}}\text{O}}_{{{\text{2max}}}}$, PPO and MMSS after 3 and 6 weeks of training. Results demonstrated that: (i) compared to CON, there was an equal speeding of $\tau {\dot{\text{V}}\text{O}}_{{2}}$ for all exercise training groups; and (ii) there were no significant correlations between speeding of $\tau {\dot{\text{V}}\text{O}}_{{2}}$ and changes in indices of performance.

*Speeding of *${\dot{\text{V}}\text{O}}_{{2}}$* kinetics after exercise training*: A novel aspect of this investigation was showing that changes in ${\dot{\text{V}}\text{O}}_{{2}}$ kinetics (i.e., the smaller $\tau {\dot{\text{V}}\text{O}}_{{2}}$ values) following exercise training were similar in all groups, independent of the markedly different intensity of exercise and metabolic disturbance at which participants trained. The observed speeding of ${\dot{\text{V}}\text{O}}_{{2}}$ kinetics occurred within the first three weeks of the intervention (i.e., PRE to MID), after which no further speeding was observed. Although this finding aligns with previous research demonstrating a rapid speeding of ${\dot{\text{V}}\text{O}}_{{2}}$ kinetics after short-term training interventions (Phillips et al. 1995; McKay et al. 2009; Murias et al. 2010, 2011a, 2016; McLay et al. 2017; Iannetta et al. 2022a), these studies had not compared how exercise intensity may have impacted this rapid response. By precisely and individually prescribing the training intensity using the intensity domain schema and matching the groups for total work (except for SIT), exercise intensity was isolated to investigate the impact of different levels of metabolic disturbance on $\tau {\dot{\text{V}}\text{O}}_{{2}}$. Thus, our study showed, for the first time, that the expected rapid speeding of ${\dot{\text{V}}\text{O}}_{{2}}$ kinetics following an exercise intervention was similar regardless of training intensity and its associated metabolic disturbance. Although young healthy individuals were investigated, results are also likely applicable to clinical populations or older individuals, where speeding of ${\dot{\text{V}}\text{O}}_{{2}}$ kinetics may be more impactful for overall health. This idea is supported by other studies that have demonstrated rapid speeding of ${\dot{\text{V}}\text{O}}_{{2}}$ kinetics in older participants (Murias et al. 2010, 2011a; McLay et al. 2017; Iannetta et al. 2022a) as well as in clinical populations (Gildea et al. 2021), but with no direct evaluation of intensity on these improvements.

That all groups exhibited a similar speeding of ${\dot{\text{V}}\text{O}}_{{2}}$ kinetics is not necessarily surprising and falls in line with previous studies that showed no impact of intensity when comparing HIIT and continuous training (Berger et al. 2006; McKay et al. 2009), or continuous training of different intensities (Murias et al. 2016). However, as mentioned, these studies utilized prescription methods that cannot ensure a homogenous metabolic disturbance across individuals. This study provides a more complete comparison of the effect of intensity by including the full spectrum of the exercise intensity domains schema through the adoption of different training modalities (i.e., interval and continuous) and with the inclusion of a control group. Thus, the present data provide strong evidence that exercise intensity does not impact the speeding of ${\dot{\text{V}}\text{O}}_{{2}}$ kinetics within the first three weeks of training, which also suggests that an “optimal” intensity for a smaller $\tau {\dot{\text{V}}\text{O}}_{{2}}$ may not exist in this sedentary/untrained population. Given that we did not evaluate more immediate effects (i.e., 1–3 days), future research could investigate whether acute sessions of different intensities impact the magnitude of decreases in $\tau {\dot{\text{V}}\text{O}}_{{2}}$. However, this is unlikely to be the case, as studies evaluating acute interventions of different intensities of continuous (Murias et al. 2016) and interval (Berger et al. 2006; McKay et al. 2009) training have shown consistently rapid speeding of the ${\dot{\text{V}}\text{O}}_{{2}}$ kinetics response.

*The connection between *${\dot{\text{V}}\text{O}}_{{2}}$* kinetics and indices of exercise performance*: It was suggested that $\tau {\dot{\text{V}}\text{O}}_{{2}}$ is an important component of fitness and performance (Jones and Burnley 2009; Rossiter 2011; Goulding et al. 2021), with evidence stemming from cross-sectional studies showing correlations between $\tau {\dot{\text{V}}\text{O}}_{{2}}$ and ${\dot{\text{V}}\text{O}}_{{{\text{2max}}}}$ (Powers et al. 1985; Zhang et al. 1991; Inglis et al. 2021b) as well as critical power (Rossiter 2011; Goulding et al. 2021). However, evidence directly comparing changes in $\tau {\dot{\text{V}}\text{O}}_{{2}}$ and performance measures in response to an exercise training intervention was lacking. When evaluating the relationship between $\Delta \tau {\dot{\text{V}}\text{O}}_{{2}}$ and $\Delta {\dot{\text{V}}\text{O}}_{{{\text{2max}}}}$, Δθ_LT_, ΔMMSS or ΔPPO at PRE to MID and MID to POST, all correlations were weak and non-significant. Together with the observation that the speeding of kinetics occurred only in the first half of the intervention while improvements in performance—measured by ${\dot{\text{V}}\text{O}}_{{{\text{2max}}}}$, θ_LT_, PPO and MMSS—were seen in both the first and second half of the intervention, these data indicate that factors other than changes in $\tau {\dot{\text{V}}\text{O}}_{{2}}$ are responsible for the improvements in these metrics.

The reasons for the disconnection between $\tau {\dot{\text{V}}\text{O}}_{{2}}$ and performance outcomes cannot be discerned from this study. A small $\tau {\dot{\text{V}}\text{O}}_{{2}}$ is undoubtedly an important component of the responsiveness of the oxidative system and helps minimize the metabolic disturbance associated with adjustments to increases in metabolic demand. However, $\tau {\dot{\text{V}}\text{O}}_{{2}}$ evaluated from moderate intensity step transitions is a response that is demonstrably easy to modify with acute and short-term interventions as supported by this and other (Phillips et al. 1995; McKay et al. 2009; Murias et al. 2010, 2011a, 2016; McLay et al. 2017; Iannetta et al. 2022a) studies. This, along with the lack of relationship between $\Delta \tau {\dot{\text{V}}\text{O}}_{{2}}$ and indices of performance, may indicate that a small $\tau {\dot{\text{V}}\text{O}}_{{2}}$ is largely a reflection of improvements in overall function within the oxygen transport cascade that result in a more efficient oxidative system (i.e., integration of central and peripheral components), rather than being directly associated with improvements in performance. In this regard, previous work has shown a disconnect between performance (i.e., ${\dot{\text{V}}\text{O}}_{{{\text{2max}}}}$) and $\tau {\dot{\text{V}}\text{O}}_{{2}}$ once a given ${\dot{\text{V}}\text{O}}_{{{\text{2max}}}}$ is achieved (Inglis et al. 2021b), suggesting that, there may be an upper limit for decreasing $\tau {\dot{\text{V}}\text{O}}_{{2}}$ values in humans, beyond which further speeding is unlikely (Inglis et al. 2021a, 2021b). In fact, it could be argued that not only elite athletes but also participants of “reasonable” (but not particularly high) fitness levels possess very fast ${\dot{\text{V}}\text{O}}_{{2}}$ kinetics (Inglis et al. 2021b) that are unlikely to become any faster, and yet they are still able to achieve improvements in performance-related variables such as those evaluated in this study via other physiological adaptations. This study, showing a dissociation between performance improvements and speeding of ${\dot{\text{V}}\text{O}}_{{2}}$ kinetics, provides strong evidence that the link between ${\dot{\text{V}}\text{O}}_{{2}}$ kinetics and performance may not be straightforward but rather coincidental (i.e., a “stronger” cardiovascular system will contribute to both a faster ${\dot{\text{V}}\text{O}}_{{2}}$ kinetics and greater performance, without one necessarily being a determinant of the other).

Interestingly, there was a reduction in ${\dot{\text{V}}\text{O}}_{{2}}$ cost of cycling at both baseline and steady state values from PRE to POST across all intervention groups; however, there was no difference in gain across any of the time points. A reduced ${\dot{\text{V}}\text{O}}_{{2}}$ at steady state is in agreement with previous research demonstrating that energetic cost of cycling can be reduced with endurance training alone in previously untrained subjects (Montero and Lundby 2015). This reduction in ${\dot{\text{V}}\text{O}}_{{2}}$ at baseline and steady-state is indicative of an improved efficiency in the ${\dot{\text{V}}\text{O}}_{{2}}$ response within the moderate-intensity domain, which could potentially result in improved performance, particularly during long duration activities. However, it is important to note that not only the speed of adjustment but also the amplitude of the ${\dot{\text{V}}\text{O}}_{{2}}$ response were not impacted by the different training interventions. Thus, this potential performance benefit would likely be associated with other physiological factors that favour a lower ${\dot{\text{V}}\text{O}}_{{2}}$ at a given intensity of exercise, rather than to the speeding effect of the ${\dot{\text{V}}\text{O}}_{{2}}$ kinetics response.

Although this study did not focus on the mechanisms that control the change in ${\dot{\text{V}}\text{O}}_{{2}}$ kinetics in response to continuous and interval endurance training, we evaluated NIRS OxCap as an indirect indicator of intracellular oxidative capacity, as this outcome might help to explain some of the mechanistic control aspects of the adaptations to exercise training. There were only weak and no significant correlations between $\Delta \tau {\dot{\text{V}}\text{O}}_{{2}}$ and ΔOxCap at the evaluated time points. This result suggests that OxCap, which is indicative of an intracellular control mechanism (Ryan et al. 2014), did not affect the ${\dot{\text{V}}\text{O}}_{{2}}$ kinetics response. However, it should be noted that this is an indirect measure of mitochondrial function, and that the lack of a significant correlation does not necessarily indicate that other intracellular control mechanisms might not be playing a key role in the response. Thus, even though we reasoned that it would be useful to explore whether $\Delta \tau {\dot{\text{V}}\text{O}}_{{2}}$ and ΔOxCap were associated, further mechanistic insights cannot be derived from this study.

## Conclusions

Speeding of ${\dot{\text{V}}\text{O}}_{{2}}$ kinetics occurs within the first 3 weeks of a six-week endurance training program and exercise intensity does not alter this outcome, even when precise intensity prescription methods are applied. Additionally, changes in $\tau {\dot{\text{V}}\text{O}}_{{2}}$ appear not to explain improvements in indices of performance including ${\dot{\text{V}}\text{O}}_{{{\text{2max}}}}$, θ_LT_, MMSS, and PPO indicating that these two outcomes are not directly connected and a coincidental association.

## Data Availability

Data displayed in this article will be made available upon reasonable request to the corresponding author.

## Abbreviations

[La^−^]: Blood lactate concentration
CON: Control
HIIT: High-intensity interval training
HRmax: Maximal heart rate
HVY1: Lower boundary of the heavy intensity domain
HVY2: Upper boundary of the heavy intensity domain
MMSS: Maximal metabolic steady state
MOD: Moderate-intensity
NIRS: Near-infrared spectroscopy
OxCap: Near-infrared spectroscopy derived oxidative capacity
P_E_CO_2_: Partial pressure of expired carbon dioxide
P_E_O_2_: Partial pressure of expired oxygen
PO: Power output
PPO: Peak power output
RCP: Respiratory compensation point
SIT: Sprint interval training
StO_2_: Oxygen saturation
${\dot{\text{V}}\text{O}}_{{2}}$: Pulmonary oxygen uptake
${\dot{\text{V}}\text{CO}}_{{2}}$: Carbon dioxide production
$${\dot{\text{V}}}_{{\text{E}}}$$: Ventilation
${\dot{\text{V}}}_{{\text{E}}} /{\dot{\text{V}}\text{CO}}_{{2}}$: Ventilatory equivalent of V̇CO_2_
VL: Vastus lateralis
${\dot{\text{V}}\text{O}}_{{{\text{2m}}}}$: Oxygen uptake the level of the muscle
${\dot{\text{V}}\text{O}}_{{{\text{2max}}}}$: Maximal oxygen uptake
$\theta_{{{\text{LT}}}}$: Estimated lactate threshold
τ: Time constant
